# Mesenchymal stromal cells derived from cervical cancer produce high amounts of adenosine to suppress cytotoxic T lymphocyte functions

**DOI:** 10.1186/s12967-016-1057-8

**Published:** 2016-10-26

**Authors:** María de Lourdes Mora-García, Rosario García-Rocha, Omar Morales-Ramírez, Juan José Montesinos, Benny Weiss-Steider, Jorge Hernández-Montes, Luis Roberto Ávila-Ibarra, Christian Azucena Don-López, Marco Antonio Velasco-Velázquez, Vianey Gutiérrez-Serrano, Alberto Monroy-García

**Affiliations:** 1 Immunobiology Laboratory, Cellular Differentiation and Cancer Unit, FES-Zaragoza, UNAM, Mexico City, Mexico; 2 Immunology and Cancer Laboratory, Oncology Research Unit, Oncology Hospital, National Medical Center, IMSS, Mexico City, Mexico; 3 Mesenchymal Stem Cells Laboratory, Oncology Research Unit, Oncology Hospital, National Medical Center, IMSS, Mexico City, Mexico; 4 School of Medicine, UNAM, Mexico City, Mexico; 5 Oriente 170 No. 160 Colonia Moctezuma 2a Sección Delegación Venustiano Carranza, 15530 Mexico City, Mexico

**Keywords:** Tumor-mesenchymal stromal cells, T cells, Adenosine, CD39–CD73, Immunosuppression

## Abstract

**Background:**

In recent years, immunomodulatory mechanisms of mesenchymal stem/stromal cells (MSCs) from bone marrow and other “classic” sources have been described. However, the phenotypic and functional properties of tumor MSCs are poorly understood. The aim of this study was to analyze the immunosuppressive capacity of cervical cancer-derived MSCs (CeCa-MSCs) on effector T lymphocytes through the purinergic pathway.

**Methods:**

We determined the expression and functional activity of the membrane-associated ectonucleotidases CD39 and CD73 on CeCa-MSCs and normal cervical tissue-derived MSCs (NCx-MSCs). We also analyzed their immunosuppressive capacity to decrease proliferation, activation and effector cytotoxic T (CD8+) lymphocyte function through the generation of adenosine (Ado).

**Results:**

We detected that CeCa-MSCs express higher levels of CD39 and CD73 ectonucleotidases in cell membranes compared to NCx-MSCs, and that this feature was associated with the ability to strongly suppress the proliferation, activation and effector functions of cytotoxic T-cells through the generation of large amounts of Ado from the hydrolysis of ATP, ADP and AMP nucleotides.

**Conclusions:**

This study suggests that CeCa-MSCs play an important role in the suppression of the anti-tumor immune response in CeCa through the purinergic pathway.

## Background

Mesenchymal stem/stromal cells (MSCs) are multipotent cells that can be obtained from various mature tissues and are characterized by a wide range of properties, including the ability to adhere to plastic; the simultaneous expression of CD73, CD90, and CD105; and the lack of or reduced expression of specific hematopoietic cell markers such as CD14, CD31, CD34, CD45 and HLA-DR [1, 2]. They can differentiate into multiple cell types, including osteocytes, chondrocytes, adipocytes, fibroblasts, myoblasts, oocytes, cardiomyocytes, hepatocytes, tenocytes, different epithelial cells (i.e., lung, intestine, kidney, and spleen), and even neurons [3–5].

MSCs may be attracted to damaged tissues by inflammatory cytokines and chemokines and support tissue regeneration and repair [6]. During this process, MSCs contact multiple cell types to carry out therapeutic effects, either by the release of extracellular vesicles (EV) expressing bioactive molecules, such as proteins, lipids, and RNA, which might play anti-inflammatory or anti-tumoral effects [7, 8], or by direct cell–cell interaction through integrins and intercellular gap junctions [9]. On the other hand, the invasive growth of tumors also causes inflammation and local tissue damage, attracting MSCs to contribute to repair [6]. Approximately 0.01 % of the cells within solid tumor tissues are thought to be MSCs (T-MSCs) [10]. These T-MSCs stimulate tumor growth by stimulating tumor cell proliferation [11], inducing epithelial–mesenchymal transition of tumor cells [12], prompting the conditions to increase the number of cancer stem cells [13], and promoting tumor angiogenesis and metastasis [6]. T-MSCs have been isolated and characterized from ovarian cancer [14], giant cell tumors of bone [15], neuroblastoma [16], osteosarcoma, lipoma [17], gastric cancer [18] and cervical cancer (CeCa) [19]. In CeCa, our research group was the first to detect the presence of MSCs and suggest their involvement in tumor cell evasion from the immune response of cytotoxic T lymphocytes [19].

In addition, T-MSCs play an important role in the immunosuppression of immune effector cells through the generation of an anti-inflammatory environment by producing Th2-type cytokines (IL-4, IL-5, IL-10) and Th3-type cytokines (TGF-β) [6, 13, 20], inducing and recruiting regulatory T-cells [21] and producing immunosuppressive factors such as PGE2, IDO and NO [22].

Recently, have been reported that MSCs derived from either bone marrow (BM-MSCs) or umbilical cord blood (UCB-MSCs), may suppress the response of T lymphocytes through the purinergic pathway [23, 24]. In this pathway, nucleotides (ATP and ADP) released by a variety of cell types in response to stress signals such as injury, hypoxia and inflammation, which commonly occur in the tumor microenvironment, are jointly hydrolyzed by CD39 ectoenzyme (ENTPD1, ectonucleoside triphosphate diphosphohydrolase-1, EC 3.6.1.5) to generate the respective nucleotides. Subsequently, the nucleotides are hydrolyzed by the action of 5′-ectonucleotidase ectoenzyme or CD73 (EC 3.1.3.5), converting AMP to adenosine (Ado) [25]. Most extracellular Ado signaling activities are mediated by G-protein-coupled cell surface adenosine receptors (ARs), which are divided into four subtypes: A1, A2A, A2B and A3 [26]. Ado may exert its immunosuppressive effect on cytotoxic CD8+ T lymphocytes (CTLs) through the high-affinity Ado receptor A2A [25, 27, 28]. High cAMP production resulting from the signaling of this receptor on T-cells inhibits the proliferation and secretion of anti-tumor cytokines, including TNF-α and IFN-γ [29, 30], as well as the exocytosis of cytolytic granules containing granzymes and perforin [31].

The expression of CD39 and CD73 in tumors is mainly induced by hypoxia [32] and by the presence of immunosuppressive factors such as TGF-β [33]. This expression is generally associated with poor prognosis [32, 34]. Persistent high-risk human papillomavirus (HR-HPV) infection plays an important role in cervical carcinogenesis, which is strongly associated with the production of immunosuppressive cytokines such as IL-10 and TGF-β. Increased IL-10 and TGF-β expression correlates directly with the degree of CeCa progression and the suppression of the anti-tumor immune response through mechanisms that have not yet been fully elucidated [35–37].

To better understand the role that MSCs play in tumor biology, in this study, we analyzed the immunosuppressive capacity of cervical cancer-derived MSCs (CeCa-MSCs) on effector T lymphocytes through the purinergic pathway. Interestingly, we found that CeCa-MSCs express higher levels of CD39 and CD73 ectonucleotidases in cell membranes compared to MSCs obtained from normal cervical tissues (NCx-MSCs). This feature is associated with the ability to strongly suppress the proliferation, activation and effector functions of cytotoxic T-cells through the generation of large amounts of Ado from the hydrolysis of ATP, ADP and AMP nucleotides. This study suggests for the first time that CeCa-MSCs play an important role in the suppression of the anti-tumor immune response in CeCa through the purinergic pathway.

## Methods

### Mesenchymal stromal cells (MSCs)

The MSCs used in this study were obtained from Normal cervix (NCx) tissues, that were obtained from three subjects who had hysterectomy surgery not related to cancer, and CeCa samples were obtained from biopsies from three patients in stage IIIB. The local ethics committee approved these procedures. The CeCa samples intended for tissue culture were collected from biopsies sent to the Pathology Department for routine diagnosis. NCx- and CeCa-derived mesenchymal stromal cells (MSCs) were obtained by enzymatic digestion as previously described [19], and maintained in low glucose Dulbecco’s Modified Eagle Medium (DMEM; Gibco Laboratories, Grand Island, NY, USA) supplemented with 15 % fetal bovine serum (FBS; Gibco), 100 IU/ml penicillin, 100 μg/ml streptomycin (Gibco) and 5 mM l-glutamine at 37 °C with 5 % CO_2_.

MSCs were characterized based on the morphological, phenotypic and differentiation parameters performed according to protocols previously reported by our working group [19]. FITC, PE or APC-conjugated monoclonal antibodies against CD73, CD90 and CD45 (BD Biosciences, San Diego, CA, USA) CD105, CD13, CD14, (Caltag, Buckingham, UK), HLA-ABC, HLA-DR, CD31 and CD34 (Invitrogen, Carlsbad, CA, USA) were used for immunophenotypic characterizations and analyzed on a CyAN cytometer (Beckman Coulter, Fullerton CA, USA). Adipogenic and osteogenic differentiation was induced with Stem Cells Kits™ (STEMCELL Technologies, Inc., Vancouver, BC, Canada). Adipogenic differentiation was determined by visualizing the presence of Oil Red O-stained (Sigma-Aldrich, St. Louis, MO, USA) lipid vacuoles. Osteogenic differentiation was assessed by alkaline phosphatase staining. Chondrogenic differentiation was induced with a commercial induction medium (Cambrex Bio Science, Walkersville, Inc., Maryland, USA) that was supplemented with 10 ng/ml of TGF-β (Cambrex). The resulting micromasses were fixed, embedded and sliced. Cross-sections were stained with Alcian blue dye (Sigma-Aldrich).

### Expression of CD39 and CD73 in MSCs

The expression of the ectonucleotidases CD39 and CD73 in MSC cell membranes was determined by labeling with anti-CD39-FITC (eBioscience, San Diego, CA, USA) and anti-CD73-PE (BD Bioscience) monoclonal antibodies according to the manufacturers’ instructions. Cell analysis was performed from the acquisition of 25,000 events in a CyAN cytometer (Beckman Coulter, Fullerton CA, USA). In addition, the expression of the ectonucleotidases CD39 and CD73 in MSCs cell membranes was also determined by immunocytochemical staining by using human anti-CD39 and anti-CD73 mAbs (Novus, Cambridge, UK). Briefly, MSCs grown on glass coverslips to semiconfluence, were fixed in 2 % paraformaldehyde (Sigma, St. Louis, MO) in PBS for 6 min and permeabilized with 0.01 % Triton X-100 in PBS. Following incubation with 2 % (w/v) bovine serum albumin (Sigma, St. Louis, MO), cells were incubated for 1 h with the mAbs. Following washing, the coverslips were incubated for 1 h with a secondary antibody horseradish peroxidase-conjugated goat anti-mouse (DAKO, Carpinteria, USA). The development was performed with substrate-chromogen solution 3,3′-diaminobenzidine dihydrochloride (DAB) for 3–5 min. Secondary antibody alone was included as control for the experiments. The nuclei were stained with Mayer’s Hematoxylin. Glass coverslips were scanned to obtain electronic files. The immunocytochemical stains of two stains made independently were digitally analyzed using the Aperio CS (San Diego, CA, USA) digital pathology equipment.

### Hydrolytic activity of CD39 and CD73

Samples containing 10^5^ MSCs were cultured in presence of 5 mM ATP, ADP or AMP in 200 µl of low glucose DMEM supplemented with 10 % FBS to determine the enzymatic activity of the CD39 and CD73 ectonucleotidases. FBS was previously dialyzed with a membrane (molecular weight cutoff of 12 kDa). To inhibit the enzymatic activity of CD39 and CD73, MSCs were incubated in the presence of 5 mM specific inhibitors, including sodium polyoxotungstate (POM-1, Sigma-Aldrich, St Louis, MO, USA) and adenosine 5′-(α,β-methylene) diphosphate (APCP, Sigma-Aldrich). After 30 min of incubation with each inhibitor, the adenine nucleotides ATP, ADP and AMP were added to a final concentration of 5 mM. The total culture volumes were 200 µl. Supernatant samples were collected every 60 min, and the presence of Ado was analyzed by thin layer chromatography (TLC) and ultra-performance liquid chromatography (UPLC) (UPLC aquity, Waters, Milford MA, USA). To analyze the samples by TLC, 1 µl of each supernatant was loaded on fluorescent plates precoated with gel (Whatman, GE Healthcare, Freiburg, Germany). Samples were eluted for 1 h using a mobile phase a mixture composed of isobutanol:isoamyl alcohol:ethoxyethanol:ammonia:water in the ratio 9:6:18:9:15 [38, 39]. ATP, ADP, AMP, Ado and inosine (Sigma-Aldrich) at 5 mg/ml were used as standards. Finally, the compounds were visualized and photographed under an UV transilluminator (UVP Biodo-H System, Upland, CA, USA).

### Ultra-performance liquid chromatography (UPLC)

An UPLC system (UPLC acquity, Waters) was used to quantify the amount of Ado generated in MSCs cultures in the presence of each nucleotide. Quantitative analysis of samples using standard quantities of synthetic Ado was carried out with Empower 3 software (Waters, USA). The mobile phase consisted of 0.5 % acetonitrile, 5 % methanol, and 94.5 % sodium acetate buffer 0.25 M, pH 6.3. Supernatant samples were centrifuged at 13,000 rpm and filtered on Amicon membranes with a cutoff of 3000 Da and subsequently diluted 1:200 with the mobile phase mixture. Run conditions were as follows: flow rate of 1.0 ml/min, UV detection at 254–260 nm, 2.0 min of retention time, room temperature, and a LiChrosfer 5 µm RP-18e 100 A (size 125 mm × 4 mm, 5 µm particle size) reverse phase column. Ado was quantified by comparing the retention time of the sample with that of the synthetic Ado used as standard.

### In vitro suppression assay for testing T cell proliferation

To determine the suppressive activity of Ado on T-cell proliferation, 1 × 10^5^ CD8+ T-cells isolated from peripheral blood mononuclear cells (PBMC) from normal donors by negative selection (EasySep Enrichment Cocktail, Stem Cell Technologies, Vancouver, BC, Canada) were cultured in triplicate in 96-well flat bottom plates (Corning, NY, USA) in 100 µl Iscove’s Modified Dulbecco’s Medium (IMDM) (Gibco) without phenol red, supplemented with 10 % dialyzed FBS. Beads coated with anti-CD2/CD3/CD28 antibodies (Miltenyi Biotec GmbH, Bergisch Gladbach, Germany) were added to T-cell cultures in a 2:1 ratio of T-cells:beads. To determine the suppressive capacity of Ado contained in the supernatants from MSCs cultured in the presence of nucleotides, 20 µl of each supernatant was added to the T-cell cultures (total volume of 200 µl) and then incubated at 37 °C and 5 % CO_2_ for 72 h. Caffeine, a nonselective antagonist of the Ado receptor, was added to some culture plates at a concentration of 300 μM (J.T. Baker, Center Valley, PA, USA). In other cases, 1 µM ZM241385, a selective antagonist of A2A receptor, or ZM241385 (1 μM) plus caffeine (300 μM) were added. Lymphocytes stimulated with beads coated with anti-CD2/CD3/CD28 antibodies alone or in the presence of synthetic Ado (500 μM), caffeine (300 μM), Ado:caffeine (500 μM:300 μM), Ado:ZM241385 (500 μM:1 μM), Ado: ZM241385:caffeine (500 μM:1 μM:300 μM) or without stimulation were seeded independently to establish appropriate controls. The CellTiter 96^®^ AQ_ueous_ One Solution (Promega, Madison, WI, USA) commercial kit was used to determine T-cell proliferation according to the procedure provided in the supplier insert. After incubating the cells for 4 h at 37 °C and 5 % CO_2_, the plate was read on an ELISA plate reader (Corning) at a wavelength of 490 nm. The percentage of T-cell proliferation was determined as follows: % proliferation = [reading T-cell cultures with conditioned media and anti-CD2/CD3/CD28 beads]/[reading T-cell cultures with anti-CD2/CD3/CD28 beads] × 100.

### Determination of IFN-γ in activated T-cells

A total of 2.5 × 10^5^ CD8+ T-cells were cultured for 48 h in a 24-well plate (Corning) with 1 ml of IMDM culture medium + 10 % dialyzed FBS in the presence of beads coated with anti-CD2/CD3/CD28 in the ratio 2:1 and in the presence or absence of 20 μL of MSCs supernatants. Caffeine (300 μM) or ZM241385 (1 μM) were added to some wells. Lymphocytes stimulated with beads coated with anti-CD2/CD3/CD28 alone or in the presence of synthetic Ado (500 μM), caffeine (300 μM), Ado:caffeine (500 μM:300 μM), Ado: ZM241385 (500 mM:1 mM), Ado: ZM241385:caffeine (500 μM:1 μM:300 μM) or without stimulus were seeded independently to establish appropriate controls. During the last 4 h of culture, Brefeldin-A (Sigma-Aldrich) was added to a final concentration of 10 μM to determine the content of intracellular IFN-γ. Subsequently, cells were collected, fixed and permeated using the Cytofix/Cytoperm Kit (BD Biosciences, San Jose, CA, USA) kit. T-cells were labeled with anti-IFN-γ/FITC and anti-CD8/APC (R&D Systems, Inc, Minneapolis, MN, USA) monoclonal antibodies, incubated for 30 min at 4 °C and washed three times with PBS. The labeled cells were evaluated in a CyAN flow cytometer (Beckman Coulter). A total of 5 × 10^4^ events were acquired to analyze the percentage of CD8+IFN-γ+ cells using Summit V4.3 software.

### cAMP assay

Measurements of cAMP levels were performed, as described previously [40]. Briefly, CD8+ T-cells (4 × 10^5^) were cultured alone or in the presence of 20 µl of each supernatant (total volume of 200 µl). In other cases, synthetic Ado (500 μM), caffeine (300 μM), Ado:caffeine (500 μM:300 μM), Ado: ZM241385 (500 mM:1 mM), Ado: ZM241385:caffeine (500 μM:1 μM:300 μM), or the ARs agonist, 5′-*N*-ethylcarboxamidoadenosine (NECA; Sigma-Aldrich) (5 μM) were seeded independently to establish appropriate controls. The cells were incubated for 30 min at 37 °C, and the reaction was stopped by addition of 1 N hydrochloric acid. cAMP levels were determined by ELISA using the Parameter™ cAMP assay kit (R&D Systems, Inc, Minneapolis, MN, USA).

### In vitro analysis of the suppression of effector CTL activity

To analyze the suppressor capacity of Ado on effector CTL function, CD8+ T-cells specific for the YMLDLQPETT peptide derived from the E7 HPV-16 protein were obtained using a method previously reported by our group [41]. A total of 1 × 10^6^ CTLs were previously cultured for 3 h in the presence of synthetic Ado (500 µM) or with 20 µl of supernatants obtained from MSCs cultured for 5 h in presence of 5 mM AMP. Subsequently, CTLs were washed and challenged with cells from the T2 lymphoblastic cell line that express empty HLA-A*02:01 molecules on their cell surfaces [42], which were loaded with 10 µM YMLDLQPETT peptide.

Target cells (peptide-loaded T2) were labeled with 5(6)-carboxyfluorescein diacetate *N*-succinimidyl ester (CFSE) (Sigma-Aldrich), and CTLs were labeled with CD8-APC antibody (BD Bioscience) following the protocol provided by BD Bioscience. The cytotoxic activity of effector cells on target cells was determined in the proportions 10:1, 5:1 and 2.5:1 (effector:target) using a cell viability solution (7AAD, Sigma-Aldrich). Viable cells were analyzed under a Coulter CyAN flow cytometer (Beckman Coulter) from the acquisition of 100,000 events by determining the percentage of positive target cells for CFSE/7AAD.

Lymphocytes were independently seeded with medium alone or medium with AMP, synthetic Ado, caffeine (300 μM), ZM241385 (1 μM), Ado:caffeine (500 μM:300 μM), Ado:ZM241385 (500 mM:1 mM) or Ado: ZM241385:caffeine (500 μM:1 μM:300 μM) to establish the appropriate controls. A total of 5 % hydrogen peroxide was used in some cases to determine the total lysis of target cells.

The percentage of lysis was calculated according to the following formula: % cytotoxicity = 100 × [(experimental lysis (CFSE+, 7AAD+) − basal lysis (CFSE+, 7AAD+)/(total lysis (CFSE+, 7AAD+) − basal lysis (CFSE+, 7AAD+)].

### Statistical analysis

All numerical data are presented as the mean value ± SEM of three independent experiments. Comparisons were evaluated by multivariate statistical analysis using GraphPad Prism version 7 (GraphPad Prism software, USA). The difference was considered statistically significant at P < 0.05.

## Results

### Immunophenotype and differentiation capacity of NCx-MSCs and CeCa-MSCs

**Fig. 1:**
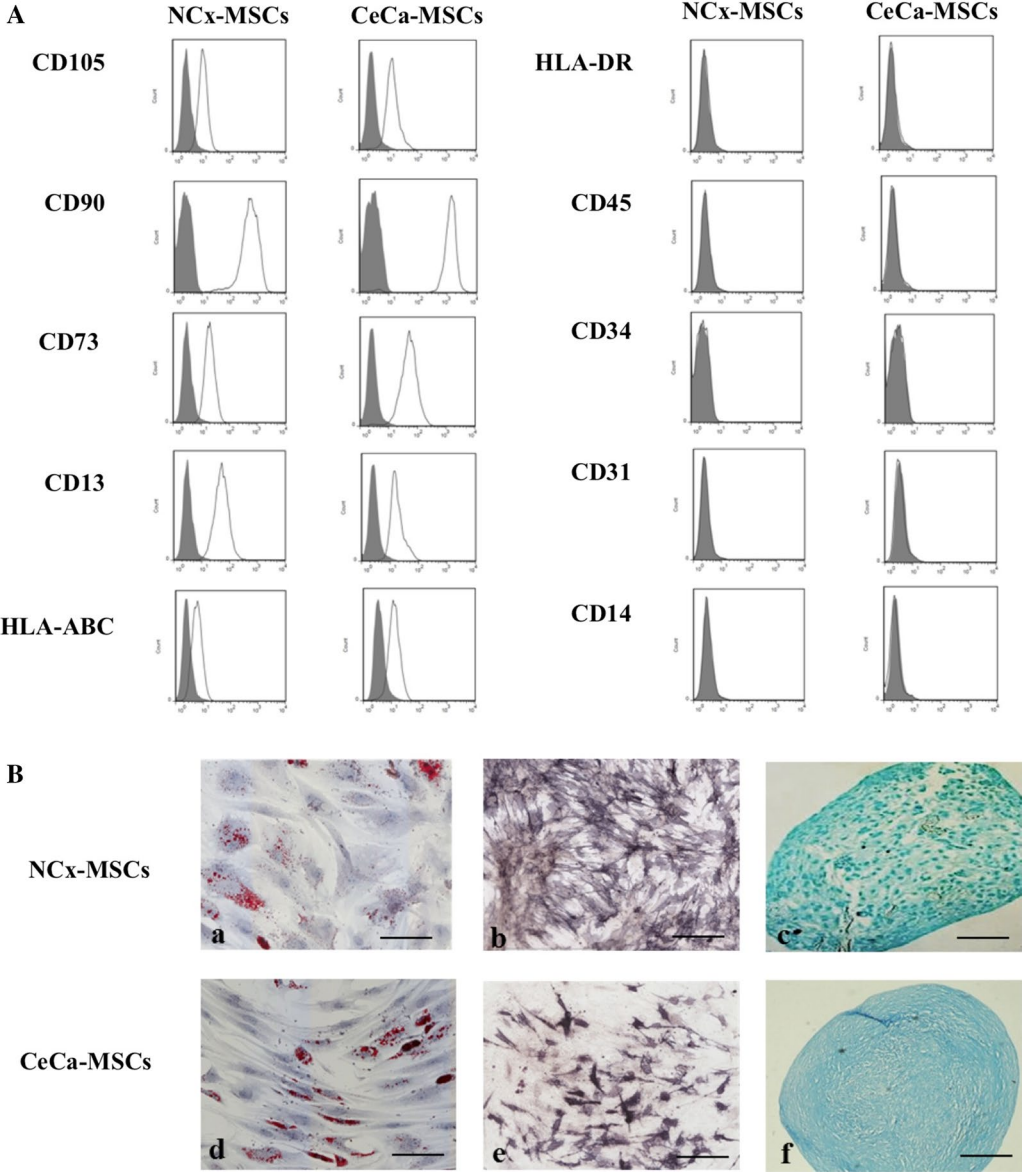
Functional characterization of mesenchymal stromal cells (MSCs) from normal cervix (NCx-MSCs) and cervical cancer (CeCa-MSCs). **A** Immunophenotype of MSCs cells. Cells were stained with FITC, PE or APC-conjugated monoclonal antibodies against surface markers indicated or immunoglobulin isotype control antibodies. Cells were analyzed using flow cytometry. Representative histograms for the indicated cell markers are shown. **B** MSCs from the two sources (NCx and CeCa) were cultured in the adipogenic, osteogenic and chondrogenic induction medium for 14, 21, and 28 days, respectively. (*a*) and (*d*) Adipogenic differentiation was indicated by accumulation of neutral lipid vacuoles that stained with Oil Red O. *Scale bar* 100 µm (magnification ×20). (*b*) and (*e*) Osteogenic differentiation was indicated with alkaline phosphatase staining. *Scale bar* 100 µm (magnification ×20). (*c*) and (*f*) Chondrogenic differentiation was indicated by the chondrogenic matrix colored by Alcian blue in cryosections from pelleted micromass. *Scale bar* 100 µm (magnification ×20). One representative experiment is showed

Individual experiments from NCx-MSCs and CeCa-MSCs displayed immunophenotypes and differentiation capacities similar to those reported previously [19]. MSCs from both sources expressed high levels of the characteristic MSCs surface markers CD105, CD90, and CD73 as established by the International Society for Cellular Therapy (ISCT) [2]. Furthermore, the MSCs expressed low levels of HLA-ABC, were HLA-DR-negative and did not express hematopoietic markers such as CD34, CD45 and CD14 or endothelial markers such as CD31 (Fig. 1A). Meanwhile, MSCs from both sources were capable of adipogenic, osteogenic and chondrogenic differentiation (Fig. 1B).

### Expression and functional activity of CD39 and CD73 ectonucleotidases in CeCa-MSCs and NCx-MSCs

**Fig. 2:**
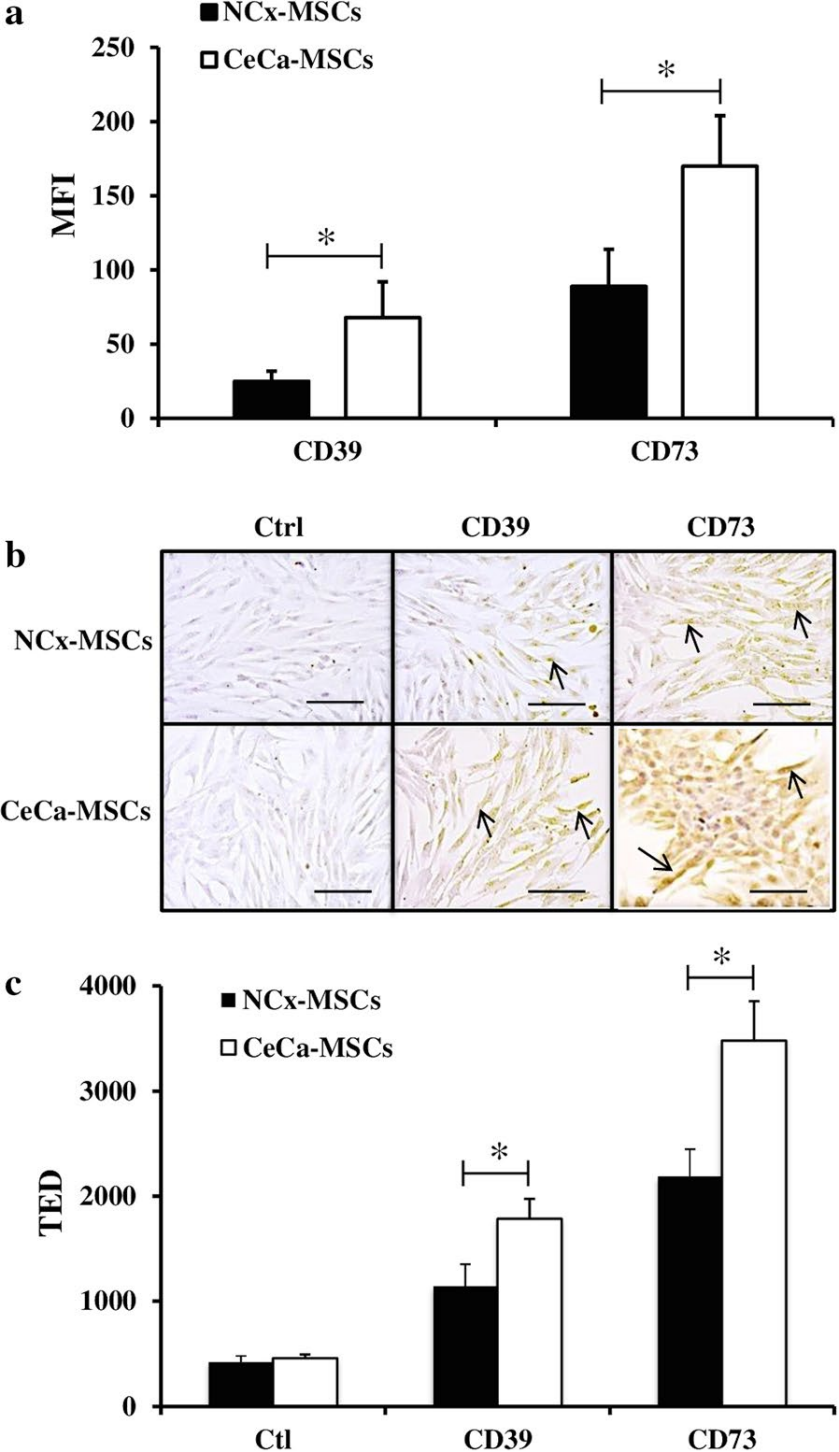
Expression of CD39 and CD73 in NCx-MSCs and CeCa-MSCs. The expression of CD39 and CD73 ectonucleotidases was determined in NCx-MSC (n = 5) and CeCa-MSC (n = 5) cell membranes by flow cytometry analysis (**a**) and by immunocytochemical analysis (**b**, **c**) as described in “Methods” section. The *arrows* indicate typical cells stained with human anti-CD39 and anti-CD73 mAbs. The mean fluorescence intensity (MFI) ± SEM of 10,000 events (**a**), and the total expression density (TED) evaluated by digital pathology using the Aperio CS system (**c**) are shown. Secondary antibody alone was included as control (Ctl) for the experiments. The images were taken at ×20 magnification (*Scale bar* 100 µm). *Asterisk* indicates significant differences (P < 0.05) compared to NCx-MSCs

In a hypoxic tumor microenvironment, Ado diminishes the ability of effector cells to kill malignant transformed cells [43]. The adenosinergic pathway contributes significantly to the immunosuppressive capacity of MSCs [44, 45]. We analyzed the expression level of CD39 and CD73 ectonucleotidases in CeCa-MSCs and NCx-MSCs, and we compared their ability to suppress the activation and effector functions of CD8+ T-cells through the production of Ado. CeCa-MSCs exhibited significantly (P < 0.05) higher CD39 and CD73 expression levels than NCx-MSCs (Fig. 2). Through flow cytometry analysis we detected that the mean fluorescence intensity (MFI) for CD39 ectonucleotidase was 68 ± 25 in CeCa-MSCs and 25 ± 7 in NCx-MSCs, and the MFI value for CD73 ectonucleotidase was 170 ± 34 in CeCa-MSCs compared to 89 ± 24 in NCx-MSCs (Fig. 2a). Similar results were observed using immunocytochemical analysis, the total expression density (TED) for CD39 ectonucleotidase was 1786 ± 189 in CeCa-MSCs and 1146 ± 206 in NCx-MSCs, and the TED value for CD73 ectonucleotidase was 3480 ± 375 in CeCa-MSCs compared to 2189 ± 258 in NCx-MSCs (Fig. 2b, c).

**Fig. 3:**
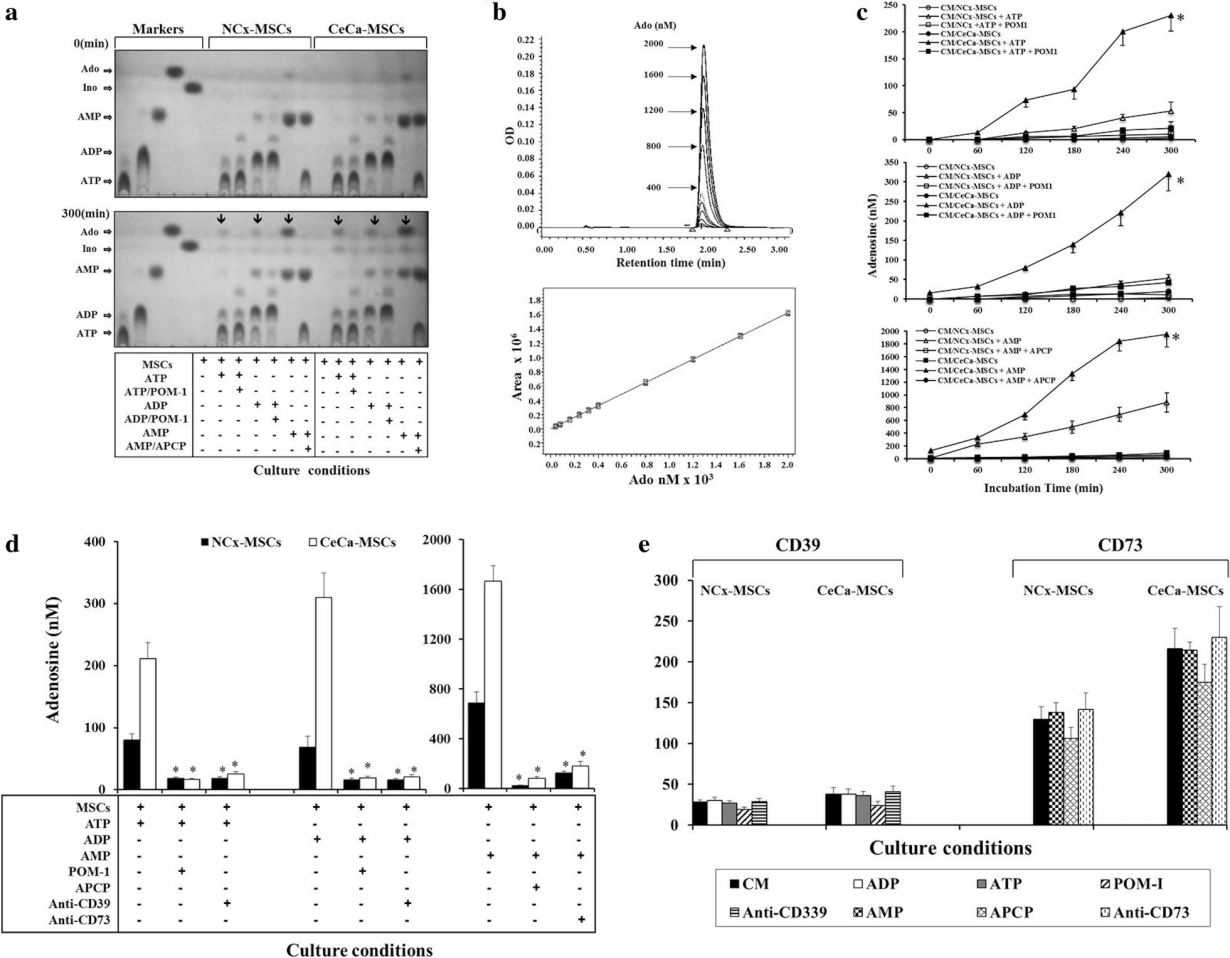
Hydrolytic activity of CD39 and CD73 ectonucleotidases expressed in MSCs. A total of 1 × 10^5^ CEMs derived from NCx-MSCs (n = 5) and CeCa-MSCs (n = 5) were cultured at 37 °C with 5 mM adenine nucleotides (ATP, ADP or AMP) in the presence or absence of POM-1 (specific CD39 inhibitor) or APCP (specific CD73 inhibitor). **a** Adenosine produced by the hydrolysis of nucleotides was analyzed by thin layer chromatography (TLC). The ATP, ADP and AMP hydrolysis products (marked with *arrows*) at the end of MSC culture with different nucleotides are shown. ATP, ADP, AMP, inosine (Ino) and synthetic adenosine were used as markers. **b** Adenosine contained in supernatant samples was quantified every 60 min by ultra-performance liquid chromatography (UPLC), using standard concentrations of synthetic Ado (*upper*). A representative linear range between concentration and histogram integral area for Ado is shown (*lower*). **c** The concentration of Ado produced by the hydrolysis of ATP (*upper*), ADP (*middle*) and AMP (*lower*) during the period of MSC culture with the respective nucleotides is shown. *Asterisk* indicates significant (P < 0.001) differences compared to NCx-MSCs. **d** The concentrations of Ado produced by the hydrolysis of adenine nucleotides (ATP, ADP or AMP) after 5 h of culture of MSCs in the presence of either ectonucleotidase specific inhibitors (POM-1 or APCP) or human mAbs (anti-CD39 and anti-CD73) are shown. *Asterisk* indicates significant (P < 0.001) differences compared to the either CD39 or CD73 basal expression. **e** The expression of the CD39 and CD73 ectonucleotidases on MSCs cultured during 5 h alone with culture medium (CM) or in the presence of nucleotides (ATP, ADP and AMP), inhibitors (POM-1 and APCP) and mAbs (anti-CD39 or anti-CD73) is shown. Data are representative of three independent experiments, and the mean ± SEM are shown

Furthermore, samples containing 1 × 10^5^ MSCs were cultured in the presence of the adenine nucleotides ATP, ADP and AMP at 5 mM to test the ability of MSCs to generate Ado through the functional activity of CD39 and CD73 ectoenzymes. Aliquots were taken from the supernatants at 60-min intervals to analyze nucleotide hydrolysis and Ado generation. TLC and UPLC were used for this analysis. According to the expression of both ectoenzymes found in MSCs membranes, after 300 min of culture in the presence of different nucleotides, CeCa-MSCs more efficiently hydrolyzed ATP, ADP and AMP nucleotides to generate Ado from each nucleotide, as shown (by arrows) in the TLC image of the products obtained after elution of the samples in TLC (Fig. 3a).

Furthermore, to determine the amount of Ado generated by MSCs during the culture period with different adenine nucleotides, aliquots were taken from the supernatants, and Ado production was quantified by UPLC. Using synthetic Ado concentrations as reference standards (Fig. 3b), the amount of Ado generated by CeCa-MSCs during the culture period was significantly higher than that produced by the NCx-MSCs (Fig. 3c). Ado concentrations in the supernatants of CeCa-MSCs cultured for 5 h in the presence of ATP, ADP and AMP were 230 ± 35, 320 ± 42 and 1950 ± 216 µM, respectively, whereas respective concentrations of 53.5 ± 12.2, 42.8 ± 6.5 and 880 ± 184 µM were found in NCx-MSC supernatants (Fig. 3c). Moreover, MSCs cultured in the absence of nucleotides showed no detectable Ado levels, and the addition of POM-1 (specific inhibitor of CD39) or APCP (specific inhibitor of CD73) decreased the ability of MSCs to hydrolyze the different adenine nucleotides by more than 90 % (Fig. 3a, c). In fact, the low Ado concentrations produced by MSCs cultured in the presence of POM1- or APCP were similar to that obtained when mAbs anti-CD39 or anti-CD73 were added to the cell cultures (Fig. 3d). Moreover, the addition of nucleotides (ATP, ADP and AMP), inhibitors (POM-1 and APCP) or mAbs (anti-CD39 or anti-CD73) to the cell cultures, did not affect the expression of the CD39 and CD73 ectonucleotidases on MSCs cell surfaces (Fig. 3e). These results provide evidence of that Ado produced by MSCs cultures in the presence of adenine nucleotides is due to the enzymatic activity of CD39 and CD73 on MSCs.

### Ado produced by CeCa-MSCs strongly inhibited the proliferation and activation of CD8+ T-cells

**Fig. 4:**
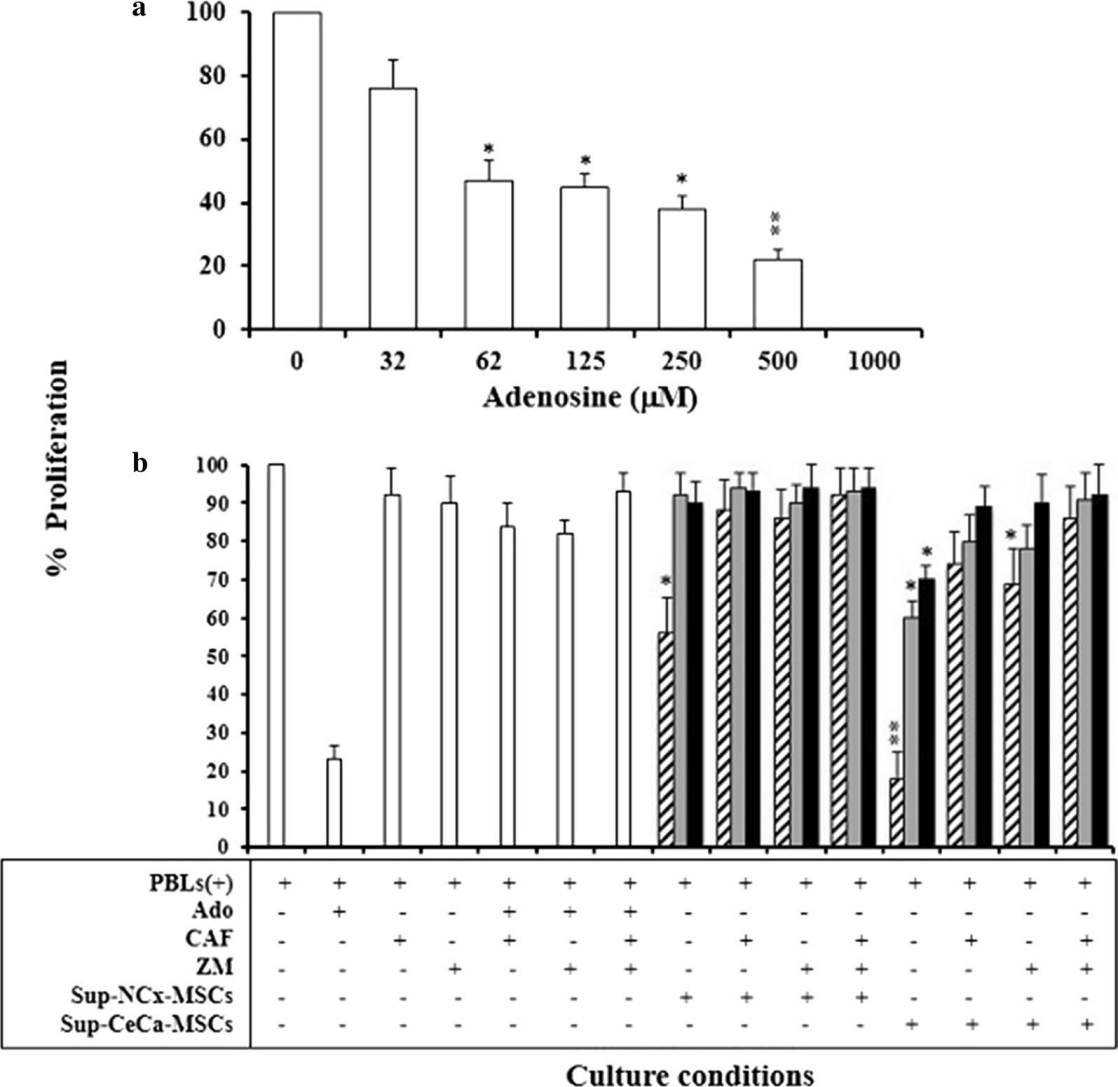
Adenosine generated by CeCa-MSCs strongly inhibits the proliferation of CD8+ T-cells. A total of 5 × 10^5^ CD8+ T-cells obtained by negative selection were cultured with beads containing anti-CD2/CD3/CD28 antibodies in a 2:1 ratio and in the presence of various concentrations of synthetic adenosine (**a**). Some cells were cultured with supernatants (Sup) obtained from NCx-MSC or CeCa-MSC cultured in the presence of the AMP (*bars with diagonal lines*), ADP (*grey bars*) or ATP (*black bars*), or in the presence of adenine nucleotides plus 300 μM caffeine (CAF), a nonselective antagonist of Ado receptors; 1 µM ZM241385 (ZM), a selective antagonist of A2A receptor, or CAF:ZM (300 μM:1 µM), as indicated (**b**). CD8+ T-cells cultured in the presence of 500 μM synthetic Ado were used as positive controls of inhibition. After 96 h of culture, CD8+ T-cell proliferation was determined by a colorimetric method described in “Methods” section. Significant differences *(P < 0.05) and **(P < 0.01) were obtained compared with CD8+ T-cells cultured with beads in the absence of Ado (negative control of inhibition). Data are representative of three independent experiments, and the mean ± SEM are shown

Ado inhibits the activation and cytotoxic effector functions of both NK cells and CD8+ T-cells [32, 38]. CD8+ T-cells were isolated by negative selection from PBMCs from normal donors to determine if the Ado generated by MSCs was able to inhibit the proliferation and activation of effector T-cells. These isolated CD8+ T-cells were cultured for 72 h with beads containing anti-CD2/CD3/CD28 antibodies. This incubation was carried out in the presence of different concentrations of synthetic Ado (32–1000 μM) and supernatants obtained from NCx-MSCs and CeCa-MSCs precultured with adenine nucleotides. Caffeine (300 μM), a nonselective inhibitor of the ARs, was added in some cultured plates. In other cases, ZM241385 (1 µM), a selective antagonist of the A2A receptor, was added. As shown in Fig. 4a, when using synthetic Ado, the inhibition of CD8+ T-cell proliferation was proportional to the increase in the concentration of synthetic Ado added to T-cell cultures. An inhibition of proliferation of 50–60 % was found at Ado concentrations of 62–250 μM. At concentrations higher than 500 μM, inhibition was greater than 80 % (Fig. 4a). NCx-MSC supernatants cultured in the presence of AMP, inhibited the proliferation of CD8+ T-cells by approximately 40 %, whereas supernatants from CeCa-MSCs doubled their inhibitory capacity, as shown (by *bars* with *diagonal lines*) in (Fig. 4b). The Ado concentration in CD8+ T-cell cultures was approximately 250 and 600 μM when adding NCx-MSC and CeCa-MSC supernatants, respectively. These concentrations were maintained in subsequent assays. Furthermore, CeCa-MSC supernatants cultured for 5 h in the presence of ATP and ADP inhibited CD8+ T-cell proliferation by approximately 30–40 %, as shown by (*bars in grey and black*) in (Fig. 4b). With supernatants derived from NCx-MSCs, the inhibition was less than 10 % (Fig. 4b). Moreover, the inhibitory effect on CD8+ T-cell proliferation was significantly blocked by adding caffeine (300 μM) or ZM241385 (1 μM) to CD8+ T-cell cultures in the presence of MSC supernatants, suggesting that the inhibition of CD8+ T-cell proliferation was due to the presence of Ado in the supernatants (Fig. 4b).

**Fig. 5:**
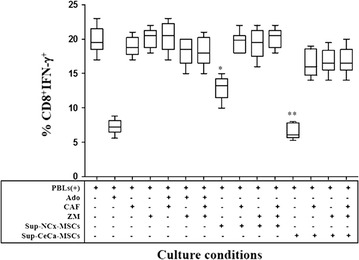
Adenosine generated by CeCa-MSCs strongly inhibits CD8+ T-cell activation. A total of 5 × 10^5^ CD8+ T-cells obtained by negative selection were cultured with beads containing anti-CD2/CD3/CD28 antibodies in a 2:1 ratio and in the presence of supernatants (Sup) obtained from NCx-MSC or CeCa-MSC cultures. Some cells were cultured in the presence of 300 μM caffeine (CAF), a nonselective antagonist of Ado receptors, or 1 µM ZM241385 (ZM), a selective antagonist of A2A receptor, and combinations of these antagonists, as indicated. CD8+ T-cells cultured in the presence of 500 μM synthetic Ado were used as positive controls of inhibition. After 96 h of culture, the CD8+IFN-γ+ T-cell percentage was determined. Significant differences *(P < 0.05) and **(P < 0.01) were obtained compared with CD8+ T-cells cultured with beads in the absence of Ado (negative control of inhibition). Data are representative of three independent experiments, and the mean ± SEM are shown

To analyze the effect of Ado on CD8+ T-cell activation, these cells were stimulated with beads containing anti-CD2/CD3/CD28 antibodies in the presence or absence of synthetic Ado or supernatants from MSCs previously cultured with AMP. After culturing cells for 48 h, we determined the percentage of CD8+IFN-γ+ T-cells. Approximately 19 ± 5 % CD8+IFN-γ+ T-cells were obtained by stimulating CD8+ T-cells in the presence of activation beads. However, 7.3 ± % of CD8+IFN-γ+ T-cells were obtained when synthetic Ado (500 μM) was added to cultured CD8+ T-cells. The percentage of CD8+IFN-γ+ T-cells obtained in the presence of NCx-MSC supernatant was 13 ± 2.5 % and in the presence of CeCa-MSC supernatant was 6 ± 1.5 % (Fig. 5). Interestingly, the addition of caffeine, the antagonist ZM241385, or both ARs antagonists, to CD8+ T-cell cultures strongly blocked the inhibitory effect of CD8+ T-cell activation produced by MSCs supernatants, suggesting that Ado generated in CeCa-MSC supernatants strongly inhibits the activation of CD8+ T-cells (Fig. 5).

**Fig. 6:**
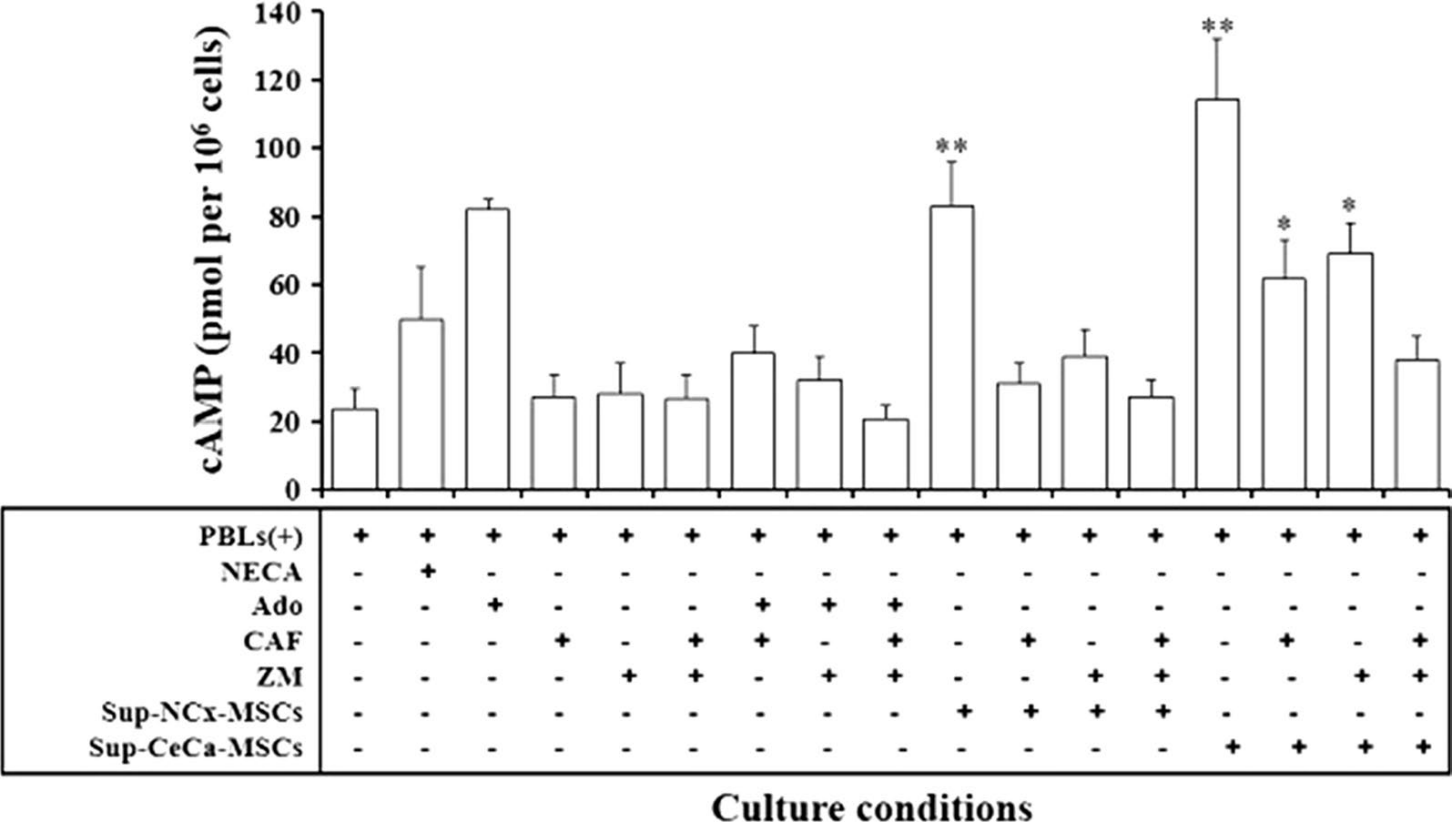
Adenosine contained in CeCa-MSC supernatants strongly increases the cAMP levels in CD8+ T cells. CD8+ T-cells (4 × 10^5^) previously stimulated with beads containing anti-CD2/CD3/CD28 antibodies in a 2:1 ratio, were cultured during 30 min in the presence of supernatants (Sup) obtained from NCx-MSC or CeCa-MSC cultures. In other cases, cells cultured in the presence of synthetic Ado (500 μM), caffeine (300 μM), Ado:caffeine (500 μM:300 μM), Ado: ZM241385 (500 mM:1 mM), Ado: ZM241385:caffeine (500 μM:1 μM:300 μM), or the ARs agonist, 5′-*N*-ethylcarboxamidoadenosine (NECA; Sigma-Aldrich) (5 μM) were seeded independently to establish appropriate controls. cAMP levels of the CD8+ T cells were determined as indicated in “Methods” section. Significant differences *(P < 0.05) and **(P < 0.01) were obtained compared with CD8+ T-cells cultured with beads in the absence of Ado (negative control of inhibition). Data are representative of three independent experiments, and the mean ± SEM are shown

Moreover, we observed that Ado generated in CeCa-MSCs supernatants was able to suppress the activation of CD8+ T cells. Thus, the addition of CeCa-MSCs supernatants to CD8+ T cells, induced a strong increase in the level of cAMP in these cells compared with the basal one, interestingly this effect was blocked by the addition of ZM241385, caffeine or both AR antagonists (Fig. 6).

### The Ado generated by the CeCa-MSCs strongly inhibits the effector function of CTLs

**Fig. 7:**
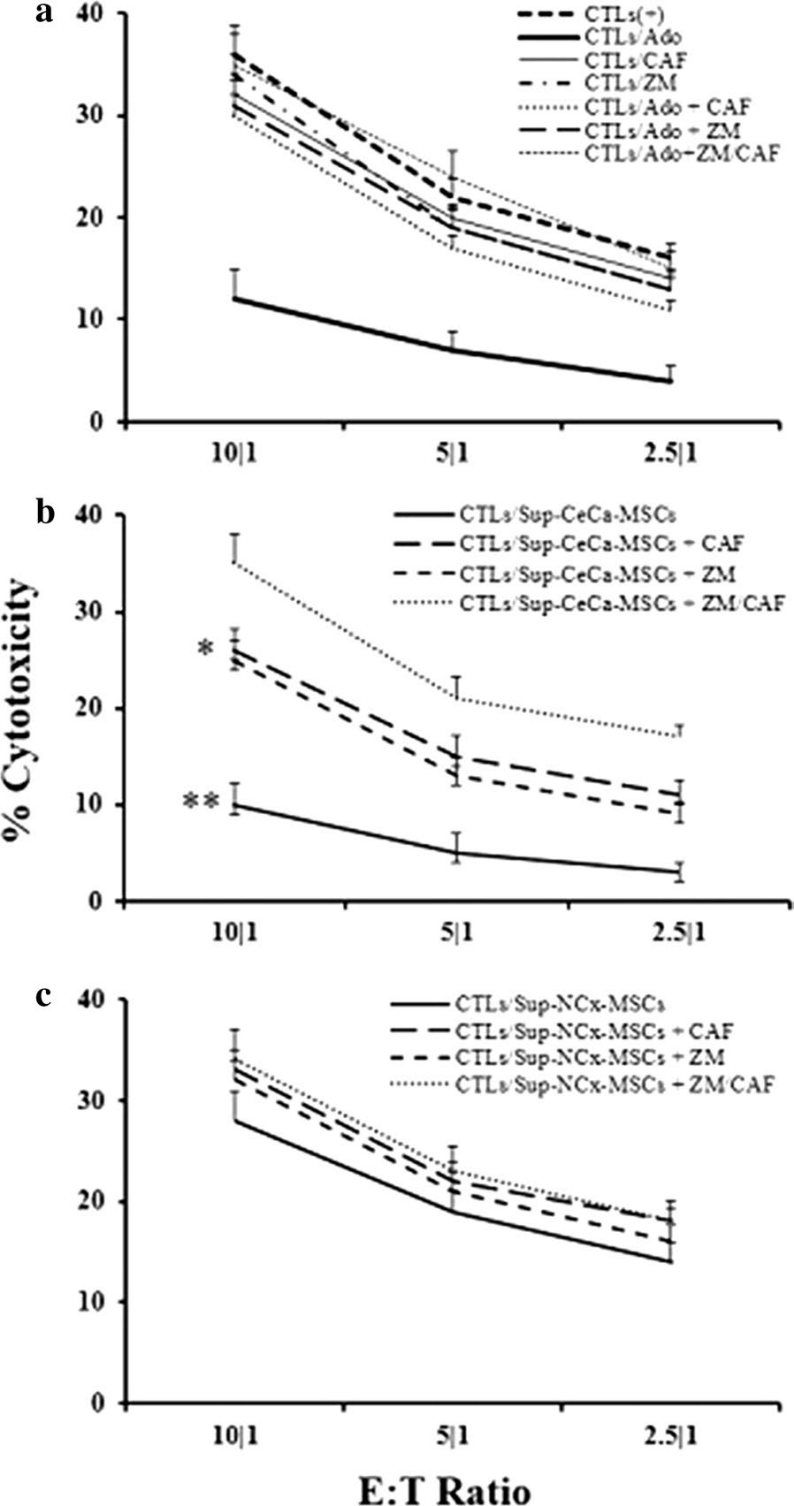
Adenosine generated by CeCa-MSCs strongly inhibits cytotoxic CTL activity. CTLs specific for the antigenic peptide YMLDLQPETT from the HPV-16 E7 protein were cultured for 3 h in the presence of synthetic 500 μM Ado (**a**) or supernatants (Sup) obtained from NCx-MSC or CeCa-MSC cultures, (**b**) and (**c**) respectively, and subsequently challenged against T2 cells loaded with the antigenic peptide YMLDLQPETT at 10:1, 5:1 and 2.5:1 ratios of effector:target cells. Some cells were cultured in the presence of 300 μM caffeine (CAF), a nonselective antagonist of Ado receptors, or 1 µM ZM241385 (ZM), a selective antagonist of A2A receptor. CTLs cultured for 3 h in the presence of 500 μM synthetic Ado were used as positive controls to analyze the inhibitory effect of Ado on CTL cytotoxic activity. The antagonists CAF and ZM were added to some culture media to block the inhibitory effect of Ado. The cytotoxic activity of CTLs was determined by a cell viability method through a CFSE and 7AAD labeling as indicated in “Methods” section. Significant differences *(P < 0.05) and **(P < 0.01) were obtained compared with the cytotoxic activity of CTLs cultured in absence of Ado. Data are representative of three independent experiments, and the mean ± SEM are shown

The previously reported [40, 46] CTL activation and CTL cytotoxic activity measurement system, was used to determine whether Ado generated by CeCa-MSCs affects the effector capacity of CTLs. CD8+ T-cells specific for the antigenic peptide YMLDLQPETT from the sequence 11–20 of the HPV-16 E7 protein with specific affinity to the HLA-A*0201 allele, were cultured for 3 h in the presence of synthetic Ado or supernatants obtained from MSCs cultured for 5 h in the presence of 5 mM AMP and subsequently challenged against T2 target cells (HLA-A*0201+) loaded with peptide YMLDLQPETT. As a positive control of inhibition, CTLs were incubated in the presence of synthetic Ado (500 μM). As expected, synthetic Ado significantly inhibited the cytotoxic capacity of CTLs (Fig. 7a). Furthermore, only CeCa-MSC supernatants comparably inhibited the cytotoxic activity of CTLs (Fig. 7b), whereas CTLs cultured in the presence of NCx-MSC supernatants showed no inhibitory effect on the cytotoxic activity of CTLs (Fig. 7c). Interestingly, the addition of either caffeine or ZM241385, or the mixture of both, to CTL cultures in the presence of synthetic Ado (Fig. 7a) or CeCa-MSC supernatants (Fig. 7b) strongly blocked the inhibitory effect of Ado on cytotoxic CTL activity. Culturing CTLs in the presence of AMP did not affect their effector activity (data not shown).

Our results strongly suggested that the differential expression of CD39 and CD73 ectonucleotidases on the membranes of CeCa-MSCs compared to NCx-MSCs may have an important role in the immunosuppressive capacity of the CTL-mediated anti-tumor immune response through the generation of Ado.

## Discussion

MSCs were described in the early 1970s in the context of regenerative medicine based on their plasticity to differentiate into chondrogenic, adipogenic and osteogenic lineages [47]. However, various immunosuppressive properties of MSCs have recently been described, paving the way for the use of MSCs in immune disorders such as graft versus host disease and multiple sclerosis [48]. Several studies have focused on elucidating MSCs involvement in malignancy development through the generation of tumors of mesenchymal origin and in the evasion and suppression of the immune system through several mechanisms that have not been fully elucidated [13, 20]. In the present study, we demonstrated that increased CD39 and CD73 expression in CeCa-MSCs allows these cells to rapidly generate Ado from the adenine nucleotides ATP, ADP and AMP, thereby exerting a strong immunosuppressive capacity on the proliferation, activation and effector functions of CTLs. In contrast, NCx-MSCs only generated Ado from AMP due to the low expression of CD39 in the membrane. In a hypoxic tumor microenvironment, Ado production is mainly mediated by the expression of CD39 and CD73 ectonucleotidases in hematopoietic and non-hematopoietic cells, including endothelial cells, immunosuppressive regulatory T-cells, mononuclear myeloid cells and tumor cells [43]. The ability of CeCa-MSCs to catalyze the ATP, ADP and AMP nucleotides and to generate immunosuppressive Ado suggests that CeCa-MSCs are part of a non-hematopoietic cell population that can significantly contribute to the generation of an immunosuppressive microenvironment. The high CD39 and CD73 expression found in CeCa-MSCs contrasts with results obtained by Saldanha-Araujo et al. [23] and Kerkelä et al. [24], who reported that both BM-MSCs and UCB-MSCs express high levels of CD73 and low levels of CD39 on their surface, enabling them to quickly generate Ado from AMP but not from ATP. However, both studies showed that the addition of activated T-cells to their culture system allowed the generation of Ado from ATP due to the high CD39 expression on activated T-cells, suggesting a co-operative functional activity between MSCs and activated T-cells in the inflammatory microenvironment to generate Ado from ATP released in the damaged tissue [23, 24]. Therefore, our results suggest that CeCa-MSCs in association with cervical tumor cells, which according to Maldonado et al. [49] and Mello et al. [50] are part of the tumor microenvironment in CeCa, can contribute significantly to the generation of Ado in the presence of high levels of ATP [49, 50].

Furthermore, adenosine produced in the tumor microenvironment inhibits the innate and adaptive immune response. The inhibitory effects of Ado on the effector cells of the immune system are mainly induced through the A2A and A2B receptors, which couple to G-proteins and induce an increase in cAMP levels, reducing the production of proinflammatory cytokines and increasing the synthesis of Th2-type cytokines and pro-angiogenic factors such as VEGF [32]. In our study, the proliferation and production of IFN-γ+ by CD8+ T-cells activated with anti-CD3/CD2/CD28 antibodies and the CTL antigen-specific effector capacity, were inhibited in a dose-dependent manner by Ado generated by MSCs. Supernatants from CeCa-MSCs contained approximately 2 mM Ado and had an inhibitory effect higher than 80 %, comparable to that observed when similar concentrations of synthetic Ado were added to cultures of activated CD8+ T-cells. In contrast, Ado levels in the NCx-MSC supernatants were less than 600 μM, with an inhibition below 20 % when added to T-cell cultures. Moreover, the use of A2A receptor antagonists was shown in recent studies to promote the anti-tumor effector activity of CTLs through a perforin-dependent mechanism, suggesting that adenosine signaling through A2A receptors inhibits the production of cytolytic granules by CTLs [51, 52]. Additionally, the antagonism of A2A an A2B receptors inhibits metastatic progression to lung in a murine model of breast cancer and is dependent on TCD8+IFN-γ+ activated T-cells, indicating that Ado signaling through these receptors also plays a role in the inhibition of effector T-cell activation [53]. In our study, we also provide evidence of that immunosuppressive activity of Ado contained in CeCa-MSCs supernatants can be through A2A receptors on CD8+ activated T cells. This is because to the fact the increased level of cAMP induced in activated CD8+ T cells by the addition of CeCa-MSCs supernatants was blocked (>80 %) by ZM241385 (selective antagonist of A2A receptor) or caffeine (adenosine analog and unspecific blocker of Ado receptors). This strongly suggests that CeCa-MSCs can participate significantly in the immunosuppression of the anti-tumor immune response through the purinergic pathway.

Based on our findings, we propose that the differential expression of CD39 and CD73 ectonucleotidases in CeCa-MSCs and NCx-MSCs, and therefore the different capacities to generate Ado, are the result of MSCs conditioning in the tumor microenvironment during the course of the disease. There is substantial evidence that BM-MSCs can migrate to the tumor, thus contributing not only to the formation of tumor stroma but also to the generation of an anti-inflammatory and immunosuppressive microenvironment [13]. For example, a study of ovarian cancer reported that 60–70 % of the cellular content of tumor stroma consisted of BM-MSCs [5]. Other studies have found that TGF-β and SDF-α cytokines as well as CXCR6 are important mediators of the migration of BM-MSCs to the tumor microenvironment [54]. Furthermore, the expression of CD39 and CD73 in the tumor microenvironment may be regulated by intrinsic physiological factors. For example, a hypoxic tumor microenvironment can promote the expression of CD73, as hypoxia-inducible factor-1α (HIF-1α) interaction with its binding site (hypoxia response element, HRE) in the CD73 gene promoter promotes CD73 expression [55]. The involvement of TGF-β in the regulation of CD39+ and CD73+ expression in various immunosuppressive cell types has also been reported. For example, CD73 expression in immunosuppressor CD4+ T-cells in mice was mainly induced by TGF-β [56]. In other studies, both myeloid suppressor cells in mouse tumors [33] and suppressor Th17 cells [57] were induced to co-express high levels of CD39 and CD73 via signaling mediated by TGF-β, therefore producing Ado as an immunosuppressive mechanism. Persistent infection with HR-HPV is associated with the production of immunosuppressive cytokines such as IL-10 and TGF-β, which increase in proportion to the degree of disease progression [35–37]. Therefore, we can hypothesize that the TGF-β produced during the development of CeCa may condition resident MSC populations or MSCs that may have migrated from the bone marrow into the tumor site to co-express high levels of CD39 and CD73 ectonucleotidases. In previous studies, we found that CeCa-MSCs induce the CeCa-derived CaSki and HeLa cell lines to increase synthesis of both IL-10 and TGF-β and to decrease expression of HLA class I molecules in the membrane, protecting these cells from immune recognition [19, 46]. Therefore, the presence of MSCs in the CeCa tumor microenvironment may favor tumor evasion from immune recognition and the immunosuppression of the anti-tumor immune response.

## Conclusions

This study found that increased expression of CD39 and CD73 ectonucleotidases in CeCa-MSC membranes compared to NCx-MSCs was associated with a strong ability to generate Ado from the hydrolysis of ATP, ADP and AMP nucleotides. In contrast, NCx-MSCs generated Ado from only AMP due to the low expression of CD39 in the NCx-MSC membranes. Interestingly, the amount of Ado generated by CeCa-MSCs cultured in the presence of adenine nucleotides significantly increased cAMP levels in activated CD8+ T cells, likewise, inhibited the proliferation, activation and effector functions of CD8+ antigen-specific T-cells. These results suggest for the first time that the CeCa-MSCs present in the tumor microenvironment may play an important role in the suppression of the anti-tumor immune response in CeCa through the purinergic pathway.

Considering that the CD39 and CD73 overexpression in tumors has been associated with a worse prognosis as well as to chemotherapy resistance in patients with several malignances [58], our results contribute in elucidate that the overexpression of CD39 and CD73 on CeCa-MSCs may support the immunosuppressive tumor microenvironment to inhibit the function of the antitumoral effector cells, such as CTLs. In consequence some therapeutic strategies should be directed to block or neutralize the functional activity of these ectonucleotidases in CeCa.

## Abbreviations

MSCs: mesenchymal stem/stromal cells
T-MSCs: tumor tissues mesenchymal stem/stromal cells
CeCa: cervical cancer
BM-MSCs: bone marrow mesenchymal stem/stromal cells
UBC-MSCs: umbilical cordon blood mesenchymal stem/stromal cells
EVs: extracellular vesicles
Ado: adenosine
ARs: adenosine receptors
CTLs: cytotoxic CD8+ T lymphocytes
HR-HPV: high-risk human papillomavirus
CeCa-MSCs: cervical cancer mesenchymal stem/stromal cells
NCx-MSCs: normal cervical tissues mesenchymal stem/stromal cells
TLC: chromatography
UPLC: ultra-performance liquid chromatography
PBMC: peripheral blood mononuclear cells
CFSE: 5(6)-carboxyfluorescein diacetate *N*-succinimidyl ester
MFI: mean fluorescence intensity
HIF-1α: Hypoxia-inducible factor-1α
HRE: hypoxia response element
